# Medical education in Latvia: an overview of current practices and systems

**DOI:** 10.3389/fmed.2023.1250138

**Published:** 2023-09-21

**Authors:** Nityanand Jain, Kirils Jersovs, Taira Safina, Mara Pilmane, Nora Jansone-Ratinika, Ieva Grike, Aigars Petersons

**Affiliations:** ^1^Faculty of Medicine, Riga Stradinš University, Riga, Latvia; ^2^Institute of Anatomy and Anthropology, Riga Stradinš University, Riga, Latvia; ^3^Center for Educational Growth, Riga Stradinš University, Riga, Latvia; ^4^Faculty of Residency, Riga Stradinš University, Riga, Latvia; ^5^Children's Clinical University Hospital, Riga, Latvia

**Keywords:** education, Latvia, curriculum development, medicine, university

## Abstract

Located in northern Europe, Latvia is one of the three Baltic States with a population of 1.9 million. The country has a rich history of medical education spanning a century and is becoming an emerging global hub for medical education. Although the surge in international students has been beneficial for the development of educational and research infrastructure, increasing demands from local students, along with institutional capacity constraints, have overburdened the available resources. Substantial investments are being made to adapt to the rapidly changing geopolitical and techno-biomedical landscape. This perspective paper presents an overview of the country’s medical education system, its challenges, and prospects from pre-university to doctoral level.

## 1. Introduction

The Republic of Latvia, with a population of approximately 1.9 million, is located on the eastern coast of the Baltic Sea in northern Europe. The country shares borders with Estonia, Lithuania, Belarus, and the Russian Federation and is one of the three Baltic States. There are 43 administrative regions in Latvia, and around one-third of the population lives in or around the capital city of Riga. According to the 2022 demographic data, the median age of the population was 42.9 years, with median ages for men and women being 39.6 and 45.7 years, respectively. The life expectancy at birth was 73.1 years (1). The difference in life expectancy between men and women is about ten years, one of the highest among all EU (European Union) countries.

## 2. Overview of Latvian healthcare system

Like many other European countries, Latvia confronts a multitude of health challenges due to a healthcare system that is highly strained and underfunded. Local experts have repeatedly stressed that the country’s performance has been poorer than the EU average in terms of access to healthcare, according to the European Commission’s European Social Policy Network (ESPN) (2). Notable health challenges for Latvia include an aging population, obesity, high tobacco and alcohol consumption, and an growing incidence of chronic diseases. The country’s demographics are strained by a shrinking population caused by high emigration rates among young people (3). The five leading reported causes of death are circulatory disorders, malignancies, COVID-19, external causes, and diseases of the digestive tract. Besides, the country has a high prevalence of traffic accidents, suicides, and communicable diseases like HIV (1, 3). Despite a reasonably comprehensive universal coverage, patients in the country still incur the second-highest out-of-pocket spending amongst EU countries. Publicly funded health services are limited by annual quotas, which often leaves patients paying for their expenses privately (4).

### 2.1. Structural Organization of the Healthcare System

The country has a publicly funded, single-purchaser healthcare system similar to the United Kingdom’s National Health Service (NHS). It offers universal health coverage to the citizens and permanent residents. The healthcare system includes both public and private players and can be broadly categorized into primary care, community and outpatient, and secondary hospital and specialist care (4). The Ministry of Health (MoH) regulates policies and regulations, while municipalities have limited responsibilities that include ensuring access, health promotion and education, and providing long-term care services. The Health Inspectorate (HI) oversees the quality of care and compliance with national regulations. There are three authorities that operate under the supervision of the MoH – the Centre for Disease Prevention and Control (CDPC; responsible for public health monitoring), the State Emergency Medical Service (SEMS; provides emergency care throughout the country), and the State Agency of Medicines (monitors safety and quality of pharmaceutical products and medical equipment).

### 2.2. Capacity and distribution of the healthcare system

The country is currently undergoing reform measures to transition from an in-patient to an out-patient care and care-at-home system (5). Consequently, the number of hospitals in the country has reduced from 65 hospitals with 11,920 beds in 2010 to 56 hospitals with a combined capacity of 9,729 beds in 2021 (1). Over the past decade, the number of healthcare institutions providing out-patient services has decreased from 4,756 in 2010 to 3,922 in 2021 (1). The country currently has 1,194 registered general medical practices, 963 specialty practices, and 457 dental practices. Also, there are 132 medical assistants (obstetrician) aid posts and 82 medical aid rooms in educational institutions across the country (1).

The healthcare system faces challenges similar to those of other countries, with a mismatch between the distribution of healthcare personnel in urban and rural areas. This imbalance sometimes leads to rural residents experiencing delays in accessing timely and necessary medical assistance (6, 7). The low wages offered in the public sector have resulted in healthcare professionals migrating to the private sector. The demographic composition of the healthcare providers (Table 1) clearly reflects the impact of an aging workforce (1, 8). Latvia has a rolling ceiling on the retirement age, which increases by 3 months every year. As of 2023, the retirement age is 64 years and 6 months.

**Table 1:** Summary data for practicing medical staff in Latvia (2015 vs. 2021).

Medical staff	Total number in 2015	Number per 10,000 inhabitants	% aged ≥ 60 years in 2015	Total number in 2021	Number per 10,000 inhabitants	% aged ≥ 60 years in 2021
Medical doctors ^1^^,^^2^	6,491	33.0	29.0%	6,328	33.7	34.0%
Dentists	1,419	7.2	21.9%	1,331	7.1	30.2%
Family doctors ^3^	1,327	6.7	30.4%	1,448	7.7	40.2%
Nurses ^4^	8,781	44.6	18.5%	7,880	42.0	23.0%

1 There are differences in the calculation methodology. Prior to 2019, the number of medical doctors included general practitioners, physicians in-service training and residents, but excluding dentists. After 2019, the number now includes the number of residents. A resident is a physician who undergoes postgraduate training in the chosen specialty under the guidance of a physician who is licensed for training according to the programme of the respective specialty provided for in the regulation for this specialty.

2 A medical doctor (or “*physician*”) is medical personnel who after a full-term study have acquired physician’s qualification and through a scientifically based medical activity directly or indirectly exerts influence upon a human being and within the framework of own professional activity examines patients to diagnose, deny, or treat a physical or mental illness. They do not include dentists, oral and maxillofacial surgeons, doctors working abroad, non-working or unemployed retired doctors, and doctors working in other sectors of the economy (administrative; research) outside the field of healthcare.

3 A family doctor (or “*general practitioner*”) is a medical doctor with additional postgraduate training in family medicine. Family medicine is the specialty in which the physician provides state-paid primary healthcare services to every individual who seeks medical care, regardless of age, gender, diagnosis, social status, etc. This includes assessing the patient’s health condition, diagnosing diseases and, within the scope of their competence, determining the necessary preventive and curative measures, as well as, if necessary, determining special examinations and the participation of other healthcare specialists in primary healthcare. The primary duty of a family doctor is to provide comprehensive and continuous healthcare.

4 There are differences in the calculation methodology. Prior to 2019, the number of nurses included roentgenology and radiology nurses (radiologist assistants). After 2019, the number now excludes dental nurses and radiologist assistants.

## 3. Higher education system

In Latvia, there are 29 higher education institutions, 16 of which are state established with remaining 13 being private. After joining the EU in 2004, the Latvian higher education underwent significant modernization to align with the principles of the Bologna process. The Latvian education system follows a three-cycle system – the first cycle leading to a bachelor’s degree, the second cycle leading to a master’s degree, and the third cycle leading to a doctoral degree. Up until October 2022, Latvia employed a credit point system, where the credit point represented an accounting unit corresponding to a student’s workload of 40 academic hours (one-week of full-time studies) (9). However, as of October 2022, the credit point system in Latvia is fully comparable to the European Credit Transfer and Accumulation System (ECTS), where 1 credit point corresponds to a workload of 25 to 30 academic hours.

## 4. Qualification regulation and accreditation

All qualifications awarded in Latvia are referenced to the Latvian Qualification Framework (LQF). The framework, developed in two phases from 2009 to 2015, is based on the European Qualification Framework (EQF), adapted to the local context and traditions. Since 2008, the Academic Information Centre (AIC) has been the nodal state government authority for the recognition of professional qualifications in regulated professions (10). The AIC is also the national coordinating body and the national EUROPASS center. AIC has also played a key role in the development of the Latvian Qualifications Database (LQD), which provides data on qualifications issued in the country since 2016. The database is updated regularly and is available in both English and Latvian (11).

The Quality Agency for Higher Education (QAHE) is a department established within the AIC that accredits higher education institutions and study programs and implements quality assurance standards. AIC is a full member of the European Association for Quality Assurance in Higher Education (ENQA), the International Network for Quality Assurance Agencies in Higher Education (INQAAHE), the Council for Higher Education Accreditation in the United States (CHEA) and is also part of the European Quality Assurance Register for Higher Education (EQAR). AIC’s extensive participation ensures the recognition and interoperability of all diplomas and qualifications issued in the country by other EU member countries. Starting in 2019, an agreement on automatic academic recognition in the Baltic States (Estonia and Lithuania) has been in effect which allows for automatic recognition of certain qualifications.

In addition to the QAHE, the Higher Education Quality Assurance Council, the Ministry of Education and Science, the Council of Higher Education (CHE), and the Latvian Council of Science are involved in the internal accreditation, licensure, framework regulation, and quality control processes. Furthermore, the Students’ Union of Latvia, Latvian Rectors’ Council, and the Employers’ Confederation of Latvia (LDDK) participate as external stakeholders in the deliberations of the internal accreditation and licensure processes (9).

## 5. Medical education in Latvia: an historical perspective

Latvia has two public universities providing medical education to both local and international candidates – the Riga Stradiņš University (RSU) and the University of Latvia (LU). Located in Riga, both universities share a centennial past. Before the 1900s, Latvians seeking medical education would travel to Estonia’s University of Tartu. However, due to a growing population and improving healthcare infrastructure, the Faculty of Medicine was established at the University of Latvia in 1919. In 1920, the first lectures were held at a former Orthodox seminary named the Theatrum Anatomicum (since it housed the Department of Anatomy).

The main faculty was selected from the midst of Latvian specialists, supplemented by international faculty from Sweden, Austria, USA, and Russian Federation in disciplines where there were no Latvian specialists available (12). Coinciding with this, in 1920, the Dentistry Department of the Faculty of Medicine was established. Later, other preclinical departments were set up at the Theatrum. At first, the curriculum mainly drew upon the Russian education system, featuring rigid departmental structures and lifelong appointment of professors. In 1922, however, a 6-year study model for medicine was implemented, followed by the establishment of the clinical base at the Pauls Stradiņš Clinical University Hospital in 1928.

In the late 1930s, the first generation of Latvia-trained doctors and professors were elected to lead the clinics and departments. After the 1940s, during the occupation of the Soviet Union, a widespread faculty replacement exercise was forced upon the staff that led to the sacrifice, repression, and emigration of many professionals. Due to suspicions regarding the university staff, including their political agendas, qualifications, and teaching models, the Stalin government officially ordered a separation of the Faculty of Medicine from the University of Latvia.

However, this plan could never be executed due to a lack of time (12). The war in the 1940s also saw the closure of the faculty for a short period followed by its restoration under the leadership of the Dean, Professor Pauls Stradiņš. The number of graduates from the medical faculty at LU was affected during the Soviet occupation period due to the re-implementation of the Soviet-style 5-year program in 1945 and its subsequent roll-back and switch to a 6-year program in 1947. Since, previous diplomas issued under German rule were not recognized by the Soviet government, many physicians were forced to retake qualification examinations in order to continue practicing medicine (12).

Finally, after several unsuccessful attempts at liquidation, the Riga Medical Institute was established in June 1950. In 1990, the Riga Medical Institute was renamed the Latvian Medical Academy. Growing contradictions inside the academy, however, led to the re-establishment of the Faculty of Medicine at LU in 1998. In 2002, the Latvian Medical Academy was re-renamed to Riga Stradiņš University, in the memory of Professor Pauls Stradiņš (1896–1958).

## 6. Pre-medical preparatory courses

Both universities provide preparatory courses to high school graduates and soon-to-be graduates as they begin planning for future medical education. Studies show that taking these courses can improve the retention of worthy candidates and create a more equal and fair selection process (13–15). Furthermore, these courses provide prospective students with an opportunity to experience the challenges and the requirements of the medical field, which can help them make informed career decisions (14) and ease their transition into medical work life. This also has been correlated with better academic performance during the early years of medical training (13, 14).

Participation in pre-medical courses in Latvia does not influence admission prospects to the same institution. The courses offer comprehensive preparation for state-administered exams in biology, chemistry, and Latvian language. RSU organizes the “*The Academy of Young Doctors*” program, which introduces high school students to a range of medical disciplines like surgery, gynecology, physiology, and gastroenterology. The program covers theoretical knowledge and practical experience through hands-on medical manipulations. Participants have the chance to participate in activities such as cardiopulmonary resuscitation, intravenous catheterization, and simulated childbirth. Similar offering by LU is titled “*The School of Young Doctors*.”

## 7. Admission to medical schools

The undergraduate medical education program in Latvia is 6 years long and leads to the award of the Medical Doctor (MD) degree (EQF Level 7). The degree corresponds to a second level professional higher education programme (360 ECTS). Local students are instructed in Latvian, although they have the option to participate in mixed groups where English is the main language. The study process for international students is conducted in English with an introductory course in Latvian language. Admission for state-funded positions is based on the overall high school grades in chemistry or biology, foreign language, mathematics, and Latvian language. Applicants can earn additional points by participating in extracurricular activities. The system is highly competitive with approximately six applicants per position. International students on the other hand must have completed their secondary education from recognized boards in biology, chemistry, English, and mathematics (Table 2). For international students, there are two intakes every year - in summer (August) and in winter (February), while for Latvian students, there is only single intake in the summer.

**Table 2:** Intake capacity for medical programme in Latvian universities.

University	Latvian students		International students
	State subsidized	Privately funded	Privately funded
University of Latvia (LU)	30 places	100 places	30 places
Riga Stradiņš University (RSU)	205 places	45 places	590 places
Total	235 places	145 places	620 places

## 8. Undergraduate medical education

Although there may be variations among university programs, for the most part they do not differ significantly. Medical education commences with an introduction to basic medical sciences (e.g., anatomy, histology, microbiology, physiology, pathology) in the first 2 years, followed by gradual introduction to clinical and research subjects (e.g., pharmacology, biostatistics, epidemiology, general surgery, cardiology) in the third year. Particular emphasis is placed on the development of communication skills relevant to the doctor-patient relationship. Training of this kind has been reported to be well received by the students as it helps them to apply their classroom knowledge more effectively in clinical settings (16, 17). Therefore, the curriculum is designed to provide early student-patient contact, including simulated and bedside interactions, from as early as the second year.

During the 4th and 5th year, the education transitions to block-based education with rotations in various clinical disciplines such as internal medicine, infectious diseases, gynecology, and surgery. Starting in 2022, the final 6^th^ year at RSU has been restructured to solely concentrate on clinical placements -17-week rotations in three departments of student’s choice. After clinical placements, the students are required to defend their research work (thesis) in a department of their preference and appear for the state examinations.

Students may select between preclinical laboratory-based research or clinical patient data-based research. Research can be completed in the form of a literature review or original data collection and analyses. As a component of the research work, students are required to independently develop their research protocol (with the guidance from their supervisor) and request the appropriate ethical approvals. The block-based system during the clinical years provides enough time for self-study and developing research directions, making it an ideal time for most students to complete their thesis. Studies indicate that incorporating a research component in medical education fosters scientific thinking and result dissemination skills among students (18–20).

### 8.1. Teaching methodologies

The students are organized into small groups of 10–12 students to ensure an interactive and personalized learning experience. Employment of such small-group teaching approaches have been reported to have positive learning benefits and outcomes (21–23). The programme places significant emphasis on fostering practical skills. During the training program, students have opportunities to improve and perfect their practical skills through hands-on training at specialized facilities. The Medical Education Technology Center (METC) at RSU has an array of diverse practical simulators, such as a laparoscopic simulator and a fully equipped simulated operating room. These resources help the students acquire and refine hands-on skills, thus enhancing their preparedness for the clinical setting. Smartboard-equipped classrooms heavily utilize visual learning aids like models, posters, and augmented reality tools.

### 8.2. Assessment methodologies

Several approaches are used to assess the acquired knowledge of medical students. Although some departments may prefer verbal evaluations, most of them favor written exams. The evaluation process generally involves the use of multiple choice questions (MCQ) along with clinical cases and open-ended questions. The education system has been swift in adapting the OSCE structure (Objective Structured Clinical Examination), particularly due to its relatively recent integration into the European national medical curricula (24, 25). Apart from theoretical knowledge, clinical skills, including the ability to gather patient data, write medical histories, formulate diagnoses, present and rationalize diagnostic hypotheses, and propose appropriate treatment plans, are also assessed to evaluate the student’s clinical proficiency.

### 8.3. Open University

Students can attend courses as non-enrolled listeners through the Open University program. The program allows individuals to choose subjects that interest them. Applicants can choose between two options – to participate as an un-enrolled listener without taking any tests or exams, or to study under the same conditions as other students and obtain a certificate. Even though admission exams are not required for participating in the Open University program, an applicant must certify that they meet the level previous education required for the chosen course.

### 8.4. Scientific interest groups and conferences

Students are encouraged to participate in different scientific focus groups, providing them with opportunities to explore and cultivate their areas of interest and deepening their knowledge (26, 27). Moreover, students receive active support for their efforts in writing and publishing scientific papers, with readily available guidance and resources to facilitate the scientific process. In addition, students have the opportunity to work in hospitals and medical institutions, such as the emergency medical service. These opportunities not only provides financial assistance but also helps students gain valuable real-world experience that can enhance their understanding of the complex and dynamic nature of the healthcare system.

LU hosts the annual “*The International Scientific Conference on Medicine*” that comprehensively explores various disciplines within clinical and research medicine, pharmacy, nursing, and public health (28). Both students and specialists are welcome to participate in this conference and share their knowledge and ideas. On the other hand, RSU, holds the biannual “*Research Week*” event, gathering various international research conferences in medicine, social sciences, public health, and university teaching and learning (29). Since 2015, as part of the Research Week, RSU has been organizing the *International Student Conference* (RSU ISC) annually. RSU ISC provides a platform for students to present their research findings and exchange ideas with fellow students as well as experts in the field. RSU ISC is the largest student-organized conference in the Baltic region, attracting a significant number of participants, with approximately 1,500 to 2000 attendees from over 30 countries.

### 8.5. International mobility and exchanges

The ERASMUS+ program provides a chance for local and international students to pursue studying abroad or complete internships with partner universities worldwide (30). The duration of the exchange can vary from a minimum of 2 months to a maximum of 12 months. To be eligible for the Erasmus program, 4th and 5th year students are expected to demonstrate proficiency in the foreign language that is required by the destination country or host institution, in addition to satisfying other prerequisites. A scholarship under the Erasmus+ grant is provided to students during the exchange, which varies in amount depending on the host country (ranging from 540 to 600 euros per month). International exchange programs under bilateral, national, and international agreements are also open to the teaching faculty. Both universities also welcome incoming students and staff members from the partner universities.

### 8.6. State examinations

The centralized examination consists of three separate components – a theoretical component, a practical component that evaluates knowledge-based practical skills, and an interpretation component that requires interaction with patients. The written theoretical component provides students with ten open questions to be answered in two blocks of 1 hour 40 minutes each. Both blocks are separated by a one hour break for the students. The exam questions cover topics from all years of medical education including basic sciences. To assess practical proficiency, students are presented with a set of five standardized tasks (manipulations) that must be completed without prior preparation. Points are individually allocated for each task, and the scores are subsequently aggregated to produce an overall score. During the interpretation component, students receive an electronic ticket which details the patient’s case subject and the medical institution where they must take the exam. The student is granted access to the patient’s data and instructed to gather the patient’s medical history within a specified timeframe using appropriate communication and patient examination skills. Afterward, the student attends a meeting with the examination committee, where they are expected to present a detailed account of their interaction with the patient.

## 9. Postgraduate medical education

Upon the successful completion of the state examinations, students have the opportunity to continue their medical education through a residency programme in more than 70 specialties, subspecialties and additional specialties.

### 9.1. Admissions to residency programme

The universities are responsible for governing the residency admission process and the implementation of training. The university is responsible for developing and maintaining the curriculum, monitoring the training process in hospitals and other medical institutions, and partially delivering theoretical education, including simulation training, administering of state exam, and granting diploma. Hospitals and other medical institutions provide practical training and partially deliver theoretical training (seminars). The duration of a residency program is regulated by the Professional Qualifications Directive 2005/36/EC and varies according to the chosen specialty, ranging from a minimum of 3 years to a maximum of 6 years. For instance, a residency in Dermatology and Venerology lasts for 3 years, whereas a residency in Neurosurgery requires 6 years. Admissions are based on a points-based system which factors various accomplishments and grades obtained during undergraduate studies. Factors such as weighted average grade and grades on the state examination, scientific work, presentations at scientific events, written scientific publications and results of an interview prior to the enrolment to the residency program in basic specialty are considered.

### 9.2. Residency programme intake capacity

The Ministry of Health determines the availability of government subsidized positions for each specialty annually, considering the demand for the specialties from hospitals, the health needs of the society, and the existing and projected supply of doctors in each specialty (31). While this planning model appears to be based on reliable indicators, realistically, the challenge to ensure a proper balance between the supply and demand remains a highly topical issue in most specialties. Consider the following example – Anesthesiologists and Reanimatologists (18 places), and Internists (20 places) have had a higher number of government subsidized study places in the last 5 years. However, the data reflects an ongoing demand for 62 Anesthesiologists and Reanimatologists and 35 Internists (32). This mismatch issue can be explained partly by the fact that for a long time the number of government subsidized residency positions were lower than the number of annual undergraduates in medicine (33).

Nonetheless, there has been an increase in the number of government subsidized positions. For the academic year 2021/2022, there were 232 available subsidized positions, which are projected to increase to 297 by the academic year 2023/2024. This increase in subsidized positions raises concerns regarding the abilities of the universities to maintain high residency training standards. This is due to the limited capacity for full cycle training process in university (tertiary level) hospitals. To accommodate the planned increase in residents intake, universities should decentralize residency training, and promote acquisition of practical knowledge in university clinics and excellence centers. This process should also focus on enhancing the skills and competences of trainees across various levels of medical institutions throughout the country.

### 9.3. Residency curriculum

The residency program provides students with the chance to gain theoretical knowledge, practical training, and hands-on experience in patient care. Furthermore, it establishes a foundation of theoretical knowledge for the state examination at the end of the residency. The curriculum and requirements of each specialty vary, for example, specialties within internal medicine such as gastroenterology, cardiology, pulmonology, and other therapeutic specialties follow a common two-year training and practice cycle module. Generally, each training and practice cycle lasts a few weeks, after which the student advances to the next cycle. Apart from the practical cycles, residents engage in theoretical seminars held by hospitals or other medical institutions, as well as other educational activities outlined in the curriculum, such as theoretical education provided by a university, pedagogical skills, and simulation training.

The curriculum is not only designed to train residents as medical experts, but also to develop interdisciplinary skills including communication, management, and research. An examination is held at the end of each educational activity. The university organizes the state examination at the end of the residency training. Besides the state examination, national legislation administers a certification examination to obtain a specialty certificate. Although legally considered separate processes, both examinations are commonly held together in most specialties. Upon completion of the residency, students become licensed specialists and commence their professional careers in their respective specialty.

## 10. Doctoral studies

The PhD programme in medicine aims to prepare highly qualified scientists and academicians. In 2022, RSU offered 36 government subsidized places and 24 paid tuition places, while LU offered 7 government subsidized places and 10 paid tuition places. Interested applicants must follow a similar admission process to that of the residency and be evaluated based on a point system. Applicants receive points for their previous scientific work and the assessment of their research plan for the intended doctoral thesis. This includes an evaluation of the written abstract and its oral defense. To complete the doctoral program, candidates must defend their dissertation before the Doctoral Committee, publicly presenting and defending their scientific paper. Successful completion of the defense leads to the conferral of the doctoral degree.

## 11. Continuing medical education

After finishing the residency programme and obtaining the specialist certificate, doctors must maintain their status as specialists by earning continuing professional development points, also known as TIP (Latvian - *tālākizglītības punktu;* English - *further education point*). The TIP system is a point-based system, that assigns points for activities such as attendance at scientific conferences, completion of educational courses, and participation in lectures. One TIP is equivalent to one academic hour or 45 min. The certificates are valid for 5 years, after which the specialists must apply for recertification.

Recertification can be obtained by accumulating 250 TIPs over 5 years, of which 150 TIPs must be obtained through professional and scientific activities related to the obtained specialty. In addition to accumulating TIPs, specialists must also provide a comprehensive account of their professional and scientific activities during the past 5 years. This account should consist of an assessment of the volume, intensity, and quality of their work, along with a detailed description of the procedures and knowledge gained during this period. This information is crucial for the recertification process, as it serves as a means to evaluate the ongoing professional development of specialists.

## 12. Challenges and future prospects

The medical education system in Latvia is being constantly evaluated, adjusted, and updated based on recent technological and biomedical advancements. For example, the current undergraduate medical education faces a number of curricular challenges as more time and attention needs to be devoted to the development of digital skills, including the use of e-medicine and telemedicine (34). The COVID-19 pandemic and lockdown have only fueled the adoption of telemedicine applications (35). Furthermore, telemedicine has been shown to be beneficial in reaching out to the patients in remote and rural areas, reducing the number of physical visits, saving time, and traveling costs, especially in patients with chronic conditions requiring frequent and multiple visits (36, 37). E-medicine is also supported by the need for reducing medical carbon footprints and adoption of green environmental practices (38). Given these advantages of telemedicine and industry-wide shifts to its adoption, it is important to train medical professionals in appropriate on-camera etiquette, background display, and body language (39, 40). Students also need to be able to understand non-verbal and verbal clues from the patient’s body language since physical examination is not possible. Finally, the ability to manage the patient examination in cases of technical glitches (patient’s or doctor’s) like camera not working are other skills that students will need training (39).

Issues of patient safety, biomedical safety, and incident reporting have also been indicated as of increasing importance in undergraduate, residency and professional development programs (41). The international political situation has also accelerated the need for extensive training in disaster and military medicine for civilian medical professionals (42–44). To improve the continuity of skill acquisition in both basic medical sciences and in clinical and research subjects, RSU has developed Skills Monitoring System that strengthens the planning, teaching, and learning, as well as the assessment phases of skill acquisition. One of the future developments will be the extension of this system beyond the undergraduate and residency study programme to the continuing education programs. Further development of simulation-based learning is another focus area as the simulation-based educational approach should be strengthened state-wide as an integral part of medical studies, supporting the transition from theory to practice, thus strengthening the quality of medical education, health care, and patient safety (45, 46). Another challenge to mention is the need to revise legislation to increase more intense implementation of simulation training and technical skills (medical methods) in the curriculum.

Given the fact that residency training is being implemented in various hospitals, post graduate medical education faces the challenge to maintain coherent training processes, including adoption of steps to prevent assigning of non-study duties to the residents due to a shortage of medical staff in the hospitals. Furthermore, more effective decentralization of practical training whilst centralization of the implementation of theoretical education, including seminars needs to be done. Research-integrated medical course leading to MD/PhD qualifications is another area for future consideration (47). Also, legislation should be reconsidered regarding the need for an officially determined joint exam at the end of residency and for the certification. Finally, the distribution model of government subsidized positions in specialties should be reviewed to ensure better matching the needs of hospitals, other medical institutions, and society as well as to the extent possible, to also provide for the wishes of the students. The implementation of medical education, which concludes with the right specialist being in the right place, should be one of the main goals for the whole system.

## Data availability statement

The original contributions presented in the study are included in the article/supplementary material, further inquiries can be directed to the corresponding author.

## Author contributions

NJ conceptualized the present study and was responsible for methodology, and software. NJ, KJ, TS, MP, IG, and NJ-R were responsible for data collection, formal analysis, investigations, visualization, and validation. NJ, KJ, TS, and NJ-R were responsible for writing the first draft of the paper. AP, MP, IG, and NJ-R were involved in revising the manuscript. MP, NJ-R, IG, and AP were responsible for supervision. All authors have read and agreed to the final version of the manuscript.

## Conflict of interest

The authors declare that the research was conducted in the absence of any commercial or financial relationships that could be construed as a potential conflict of interest.

## Publisher’s note

All claims expressed in this article are solely those of the authors and do not necessarily represent those of their affiliated organizations, or those of the publisher, the editors and the reviewers. Any product that may be evaluated in this article, or claim that may be made by its manufacturer, is not guaranteed or endorsed by the publisher.
